# Assessing Crop Coefficients for Natural Vegetated Areas Using Satellite Data and Eddy Covariance Stations

**DOI:** 10.3390/s17112664

**Published:** 2017-11-18

**Authors:** Chiara Corbari, Giovanni Ravazzani, Marta Galvagno, Edoardo Cremonese, Marco Mancini

**Affiliations:** 1 Department of Civil and Environmental Engineering, Politecnico di Milano, Piazza Leonardo da Vinci, 32, 20133 Milan, Italy; giovanni.ravazzani@polimi.it (G.R.); marco.mancini@polimi.it (M.M.); 2 Environmental Protection Agency of Aosta Valley, Climate Change Unit, 11100 Aosta, Italy; m.galvagno@arpa.vda.it (M.G.); e.cremonese@arpa.vda.it (E.C.)

**Keywords:** crop coefficient, natural vegetated area, satellite data, eddy covariance stations

## Abstract

The Food and Agricultural Organization (FAO) method for potential evapotranspiration assessment is based on the crop coefficient, which allows one to relate the reference evapotranspiration of well irrigated grass to the potential evapotranspiration of specific crops. The method was originally developed for cultivated species based on lysimeter measurements of potential evapotranspiration. Not many applications to natural vegetated areas exist due to the lack of available data for these species. In this paper we investigate the potential of using evapotranspiration measurements acquired by micrometeorological stations for the definition of crop coefficient functions of natural vegetated areas and extrapolation to ungauged sites through remotely sensed data. Pastures, deciduous and evergreen forests have been considered and lower crop coefficient values are found with respect to FAO data.

## 1. Introduction

Accurate assessment of evapotranspiration is fundamental for efficient water resource management, for the design and management of water supply reservoirs, for designing and scheduling irrigation systems, and for environmental assessment [1]. Moreover, trends of modifications of water availability around the world, have prompted scientific investigations of the effect of climate change on the hydrological cycle [2,3,4,5]. In order to study the effects of climate change for several years in advance, the use of parsimonious models for the assessment of evapotranspiration is needed in order to maintain the simulation time within acceptable limits and because of limited availability of meteorological data at a spatial and temporal resolution sufficient to accurately capture the dynamics of hydrological processes.

Latent heat flux (LE), i.e., evapotranspiration (ET), is the joint term that rules the relationship between water and energy fluxes through vegetated soil and shallow atmosphere layer, since it is the only term present both in the water as well as in the energy balance. Despite its important role, direct measurements of ET are neither frequent nor simple, due to the complexity of the process, its spatial scale of representativeness and plant phenology. These issues often affect the reliability of direct measures of water losses, so that the scientific community proposed in the late forties onwards several methods to estimate ET based on meteorological measures [6]. A simplified method is based on the crop coefficient (kc) which embodies all the physiologic characteristics of a specific plant allowing one to relate a reference evapotranspiration of well irrigated grass to the potential evapotranspiration of each crop [7]. The kc is affected by growth stage, crop types, canopy conductance, soil characteristics, crop height and leaf area index.

This approach, which has been adopted by the Food and Agricultural Organization (FAO), is based on a revised version of the Penman-Monteith equation. Alternatives were proposed for reference evapotranspiration assessment that use Hargreaves-Samani equation [8] and its derivatives [9,10,11].

A database of kc values for a large number of agricultural crops has been created by [7] for different climates and [7] presented procedures for estimating kc values for natural vegetation as a function of leaf area index. However, local ground measurements are still needed to estimate a local corrected kc to account for local crop varieties and for specific weather conditions [12].

Potential evapotranspiration, which was usually measured with a weighting lysimeter as described in FAO paper no. 56, suffers from important limitations, and can also be defined with more innovative techniques. In particular micro-meteorological stations, based on eddy covariance, are regarded to be accurate enough for ET estimates and are now widely diffused [13,14,15,16] in cultivated fields as well as in natural vegetated areas.

Many works have been done in the last decades in order to calculate the crop coefficients in specific areas (more numerous in semi-arid and arid regions) for a large variety of crops [17,18,19,20,21]. The kc in these papers are locally obtained from lysimeter or eddy covariance measurements and result, in general, values that are lower than the FAO paper no. 56 ones.

Although the kc method has been widely used for crops, it has not been widely examined for natural ecosystems, such as forests and other perennial vegetation [22,23]. A few examples are available from [24] who analyzed small shrubs founding a kc between 0.50 and 0.85 while for well-developed shrubland a kc between 0.85 to 0.95 was determined. Reference [23] analyzed different vegetation covers obtaining that, except for evergreen forests, kc values have large seasonal variation as a function of crop growth and mainly precipitation.

Moreover in the last few years remote sensing data are also always more frequently used in support of evapotranspiration estimates defining vegetation parameters and crop coefficients in a distributed way for agricultural crops with empirically- or physically-based relationships [25,26,27,28,29,30]. The greater advantage of the use of remote sensing data is the possibility to have spatially distributed information. In particular, methods that combine vegetation index and crop coefficients have been developed in agricultural crops from 30 years ago [31].

Two different objectives are defined in this paper:

(a) The definition of crop coefficient curves for natural area derived from eddy covariance data to be used in hydrological modelling to compute effective evapotranspiration.
(b) Assessing the reliability and potentiality of using satellite data and conventional meteorological measurements for crop coefficient estimates in natural vegetated areas where eddy covariance stations are not available.

In this paper the proposed procedure is applied for three types of natural coverage: pastures, deciduous and evergreen forests.

## 2. Materials and Methods

Much literature discusses “potential” and “actual” ET, the former being the maximum evapotranspiration which would occur if no factors other than energy supply and atmospheric demand limiting the ET rate [32]. Conversely, “actual” ET is the amount of water evapotranspirated in the case that water availability becomes a limiting factor [33].

Potential evapotranspiration can be derived from ground measurements, lysimeter or eddy covariance stations, or computed using a wide range of equations that are very well assessed in the research community, such as the Prietsley-Taylor equation [34], Hargreaves [8] or Penman Monteith [6]. In this study the Penman-Monteith equation has then been applied.

### 2.1. Penman-Monteith Equation

Penman-Monteith’s equation, that combines the energy balance with the mass transfer method, allows computing a potential evapotranspiration (*ETP_PenMon_* (mm·day^−1^) as:

(1) $$ETP_{PenMon}=\frac{\left[\Delta (R_{n}-G)+c_{p}\rho_{a}(e_{0}-e_{a})/r_{a}\right]}{\Delta +\gamma \left(1+r_{c}/r_{a}\right)}$$

where *R_n_* is the net radiation (MJ·m^−2^·d^−1^), *G* is the soil round heat flux (MJ·m^−2^·d^−1^), (*e*_0_ − *e_a_*) is the vapour pressure deficit of the air (kPa), *ρ_a_* is the mean air density (kg·m^−3^), *c_p_* is the specific heat of the air (MJ·kg^−1^·°C^−1^), Δ is the slope of the saturation vapour pressure (kPa·°C^−1^), *γ* is the psychrometric constant (kPa·°C^−1^) and *r_c_* is the crop resistance (s·m^−1^) and *r_a_* is the aerodynamic resistance (s·m^−1^).

In particular net radiation is computed as sum of longwave and shortwave radiation:

(2) $$R_{n}^{}=(1-\alpha )\times R_{s}-R_{nl}^{}$$

where *α* is albedo, *R_s_* is the incoming shortwave radiation (MJ·m^−2^·d^−1^) and *R_nl_* is the net longwave radiation (MJ·m^−2^·d^−1^).

The aerodynamic resistance is a function of wind velocity (*u*) (m·s^−1^) and canopy height (*h_c_*) (m) [35]:

(3) $$r_{a}=\frac{\ln\left(\frac{z_{u}-0.667h_{c}}{0.123h_{c}}\right)\ln\left(\frac{z_{t}-0.667h_{c}}{0.0123h_{c}}\right)}{0.168\times u}$$

where *z_u_* and *z_rh_* are respectively the measurement heights of wind velocity and relative air humidity (m).

The crop resistance is computed as [36]:

(4) $$r_{c}=\frac{r_{s\min}}{0.5\times LAI}$$

where *r*_*s*min_ is the minimum stomatal resistance (s·m^−1^) and *LAI* is leaf area index. *G* is approximated as a fraction (0.1 during daytime [7]) of net radiation.

### 2.2. Sensitivity Analysis

A sensitivity analysis on kc is performed to understand how much it deviates from its mean value. A sensitivity coefficient is computed in respect to kc for each parameter included in crop coefficient estimates (e.g., LAI, albedo and vegetation height) and then is adimensionalized so that the results are comparable. The coefficient of sensitivity is computed as:

(5) $$S_{Kc}=\underset{\Delta kc\to 0}{\lim}\left(\frac{\Delta V_{i}/V_{i}}{\Delta kc/kc}\right)=\frac{\partial V_{i}}{\partial kc}\frac{kc}{V_{i}}$$

where *S_Kc_* is the sensitivity coefficient and *V_i_* is the *i*th parameter. A positive value of *S_Kc_* means that the parameter will increase as kc increases and the opposite behaviour for negative values of *S_Kc_*.

### 2.3. Eddy Covariance Technique

The eddy covariance method determines the surface fluxes as the sum of turbulent fluxes, measured above the surface, and the flux divergence between the surface and the eddy covariance measurement level [37]. The basic equations to estimate latent and sensible heat fluxes are:

(6) $$LE=\lambda \rho \bar{w\prime H_{2}O\prime }$$

(7) $$H=\rho C_{p}\bar{w\prime T\prime }$$

where *λ* is the vaporization latent heat, *ρ* the air density and $\bar{w\prime H_{2}O\prime }$ the covariance between vertical wind velocity and concentration of vapor in the air. *C_p_* is the specific heat at constant pressure and $\bar{w\prime T\prime }$ is the covariance between vertical wind velocity and temperature. The symbol (ʹ) indicates instantaneous fluctuation from the time-averaged values of a specific variable in according with Reynolds decomposition of a meteorological stochastic signal [38].

The reliability of flux measurements depends on different theoretical assumptions of the eddy covariance technique [17,39], among which the most important are the horizontal homogeneity, the stationarity and the mean vertical wind speed equal to zero during the averaging period. A number of corrections needs to be applied to obtain high quality fluxes flowing procedures which are now well assessed in literature [40,41].

### 2.4. The FAO Crop Coefficient

According to FAO Paper no. 56 [7], the crop coefficient is given by:

(8) $$kc=\frac{ETP}{ET_{0}}$$

where *ET*_0_ is the reference potential evapotranspiration (mm·day^−1^) and *ETP* is a generic potential evapotranspiration (mm·day^−1^). According to FAO Paper no. 56, three values of kc are defined over time in accordance to the crop development stage: (i) initial stage (*kc_-ini_*), (ii) mid-season stage (*kc_-mid_*) and the harvest stage (*kc_-end_*).

The reference evapotranspiration, *ET*_0_, is a potential evapotranspiration which derives from Penman-Monteith’s equation and is defined for a reference surface defined as an “hypothetical crop with an assumed height of 0.12 m having a surface resistance of 70 s·m^−1^ and an albedo of 0.23, closely resembling the evaporation of an extension surface of green grass of uniform height, actively growing and adequately watered” [7].

*ET*_0_ (mm·day^−1^) is computed as:

(9) $$ET_{0}=\frac{0.408\Delta (R_{n}-G)+\gamma \frac{900}{T+273}u_{2}(e_{0}-e_{a})}{\Delta +\gamma (1+0.34u_{2})}$$

where $u_{2}$ is wind speed at 2 m above ground surface (m·s^−1^) expressed as:

(10) $$u_{2}=\frac{4.87}{\ln(67.8z_{u}-5.42)}u$$

For natural vegetation [7] proposed to assess crop coefficient as a function of leaf area index (LAI)

(11) $$kc_{FAO}=k_{c,\min}+\left(k_{c,\max}-k_{c,\min}\right)\left(1-e^{-0.7LAI}\right)$$

where:

(12) $$k_{c,\max}=k_{c,h}+\left[0.04\left({\bar{u}}_{2}-2\right)-0.004\left(RH_{\min}-45\right)\right]{\left(\frac{h}{3}\right)}^{0.3}$$

(13) $$k_{c,h}=1+0.1h$$

where ${\bar{u}}_{2}$ is the mean value for wind speed at 2 m height during the mid-season (m·s^−1^), $RH_{\min}$ is the mean value for minimum daily relative humidity during mid-season (%), and *h* is the mean maximum plant height (m).

### 2.5. Crop Coefficients

In this paper, crop coefficient is assessed in three ways that are different as regards ETP estimates:

(14) $$kc_{eddy}=\frac{ETP_{eddy}}{ET_{0}}$$

(15) $$kc_{PenMon,sat}=\frac{ETP_{PenMon,sat}}{ET_{0}}$$

(16) $$kc_{PenMon,ground}=\frac{ETP_{PenMon,ground}}{ET_{0}}$$

where *ETP_eddy_* is the potential evapotranspiration derived by eddy covariance stations, *ETP_PenMon,sat_* is the potential evapotranspiration estimated with Penman-Monteith’s equation using leaf area index and albedo retrieved from remotely sensed images and ground measured meteorological forcings, and *ETP_PenMon,ground_* is the potential evapotranspiration estimated with Penman-Monteith’s equation using data coming from ground measurements.

Since the crop coefficient approach proposed by FAO is intended for potential evapotranspiration estimate, *kc_eddy_* is significant only when vegetation can evapotranspirate with no limitation in unlimited water conditions. As it can be expected that, when available moisture of the soil is enough, ET from eddy covariance station should be similar to the one calculated with the Penman-Monteith’s equation (Equation (1)), in the present work *kc_eddy_* is computed only when the condition $\frac{ETP_{eddy}}{ETP_{PenMon,ground}}\approx 1$ (±0.05) is satisfied.

**Figure 1:**
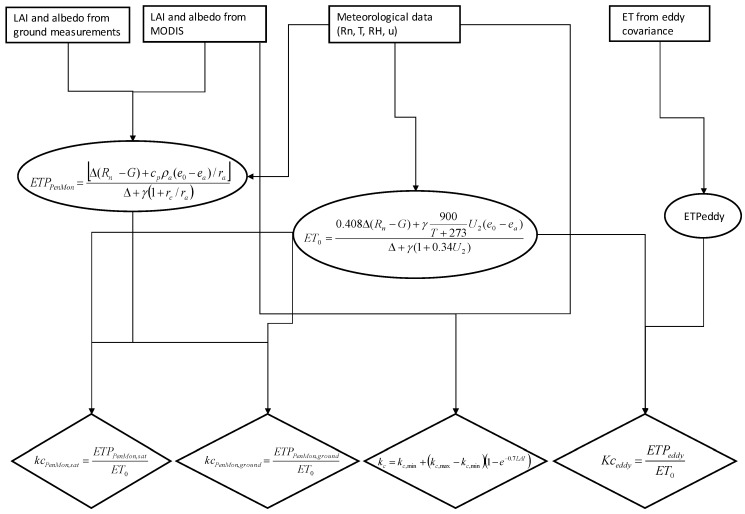
Methodology to compute crop coefficients with the four different techniques.

In addition to above mentioned methods for crop coefficient assessment, Equation (12) is applied (*kc_FAO_*). A schematic of the methodology followed in this research is illustrated in Figure 1.

### 2.6. Sites and Data

Monitoring took place in four sites representative of three natural ecosystems: the first one is located in a grass field in the mountain area of Torgnon in Italy [45°50′40′′ N, 7°34′41′′ E, 2160 m above sea level], the second, Chestnut Ridge in the U.S. [35.9′ N, 84.3′ W, 286 m a.s.l.], and the third, Duke Forest in the U.S. [35.9′ N, 79.1′ W, 168 m a.s.l.], are located in the deciduous broadleaf forest region, while the fourth is in the Black Hills in the U.S. [44.1′ N, 103.6′ W, 1718 m a.s.l.] characterized by an evergreen forest.

#### 2.6.1. Torgnon

The study site of Torgnon [42,43] is an abandoned pasture located in northwestern Italian Alps (Aosta Valley, Italy) at an elevation of 2160 m a.s.l. The site is part of the FLUXNET network. This site is characterized by an intra-Alpine semi-continental climate, with an annual mean temperature of 3.1 °C and a mean annual precipitation of about 880 mm. From the end of October to late May, the site is covered by a thick snowcover (90–120 cm) which limits the growing period, and hence plants evapotranspiration, to an average of five months. Dominant vegetation is composed by matgrass (*Nardus stricta* L., *Festuca nigrescens* All., *Arnica montana* L., *Carex sempervirens* Vill., *Geum montanum* L., *Anthoxanthum alpinum* L.L., *Potentilla aurea* L., *Trifolium alpinum* L..) and the soil is classified as Cambisol (WRB classification).

The eddy covariance method is used to measure the fluxes of H_2_O between the ecosystem and the atmosphere. Measurement of wind speed in the three components is performed by a CSAT3 three-dimensional sonic anemometer (Campbell Scientific, Logan, UT, USA), while H_2_O vapor air densities were measured by a LI-7500 open-path infrared gas analyzer (LICOR, Lincoln, NE, USA). Instruments were placed at 2.5 m above the ground. Data from both the anemometer and the IRGA were measured at 10 Hz. A weather station is installed close to the eddy covariance tower to continuously measure additional meteorological variables. Air and soil temperatures are measured respectively by a HMP45 (Vaisala, Helsinki, Finland) and with temperature probes type therm107 (Campbell Scientific) at different depths. Soil water content is assessed with soil water reflectometers model CS616 (Campbell Scientific). The variables of interest for this study, i.e., soil heat flux and net radiation are measured respectively by HFP01 plates (Hukseflux, Delft, The Netherlands) and by a CNR4 (Kipp & Zonen, Delft, The Netherlands) net radiometer. Snow height is measured with a SR50A sonic snow depth sensor (Campbell Scientific).

Since the grassland is unmanaged, canopy structure varies during the season following the phenological development. Measurements of canopy height and Leaf Area Index (LAI) at site are available for the study years, in detail: canopy height is measured and phytomass is sampled every two weeks at 12 selected plots of 40 cm × 40 cm in the study area. Total LAI and canopy height for the 12 plots are averaged to obtain a site-level value.

#### 2.6.2. Chestnut Ridge

Chestnut Ridge is a site included in the FLUXNET network (ORNL DAAC, 2011) located in Tennessee (USA) in a temperate climate and managed by Oak Ridge National Laboratory in Tennessee. The vegetation type below the station is deciduous broadleaf forest and in particular oaks and hickories are present. The tower height is 60 m. The station is active from 2006 and the data analysed in this paper are from the year 2007.

The micro-meteorological station is supplied with the following sensors: a Young-81000 3D sonic anemometer (Young, Traverse City, MI, USA), a LI-7500 open path gas analyzer (LICOR), a CNR1net radiometer (Kipp & Zonen), a Gcap Temperature/Humidity Probe (ATDD/NOAA, College Park, MD, USA), soil moisture sensor delta-T probes at different depths, Soil Temperature Probe and a HFP01heat flux plate (Hukseflux) and a TB3 Tipping Bucket Rain Gauge (Hydrological Services, Lake Worth, FL, USA). Data are available every 30 min. A more detailed description of this station can be found in [44,45].

#### 2.6.3. Duke Forest

The Duke Forest C-H2O Research Site is located at the Blackwood Division of the Duke Forest near Durham (North Carolina, USA) and is part of the FLUXNET network. The forest is characterized by oak-hickory trees of about 33 m. The tower, which is 40 m tall, is equipped with a CSAT3 3D sonic anemometer (Campbell Scientific), a LI-6262 open path gas analyzer (LICOR), a NR-LITE net radiometer (Kipp & Zonen), a HMP35C Temperature/Humidity Probe (Vaisala) and soil moisture sensor delta-T probes at different depths. Data are available every 30 min and data from 2001 and 2002 are used in this paper. The papers that describe the setup of the station are [46,47].

#### 2.6.4. Black Hills

The Black Hills eddy covariance station is located in South Dakota (USA) in a temperate climate and is managed by Oak Ridge National Laboratory in Tennessee (USA). It is part of the FLUXNET network. The tower is 24 m height and the vegetation coverage is composed by evergreen needleleaf forest, in particular ponderosa pines. The station is equipped with a R3sonic anemometer (*Gill*), a LI-7500 open path gas analyzer (LICOR), a CNR1 net radiometer (Kipp & Zonen), a HMP50Y Temperature/Humidity Probe (Vaisala), a HFP01 soil heat flux plate (Hukseflux), a ThetaProbe ML2 soil moisture sensors (Delta-T, Cambridge, UK) and soil temperature probes (ATDD/NOAA). A more detailed description of this station can be found in [47]. Data are available every 30 min and data from 2006 and 2007 are used in this paper.

#### 2.6.5. Satellite Data

Leaf area index and albedo are retrieved from satellite data measured from MODIS radiometers on board TERRA and AQUA NASA satellites (https://earthdata.nasa.gov/). These satellites fly over the same area twice a day, providing a night and a day image. The values of LAI [48] and albedo [49] are available products which are averaged over a range of eight days to avoid problems of cloudy conditions. These parameters can be considered constant during this period of time.

## 3. Results and Discussion

kc values are computed at daily scale from measured eddy covariance data at 30 min or 1 h. A day of data is considered only if at least the 80% of daytime data is available (when *R_n_* > 0), otherwise the entire day is deleted. Other gaps are also present in the observed series due to the identification of potential evapotranspiration conditions. In fact days characterized by soil water stress conditions are eliminated from the data series.

Finally in order to compute the difference between the computed crop coefficient values, RMSE is evaluated as:

(17) $$RMSE={\left[\frac{{\sum_{i=1}^{n}\left(X_{sim}{}_{}^{i}-X_{obs}{}_{}^{i}\right)}^{2}}{n}\right]}^{0.5}$$

where *X_sim_^i^*^th^ is the *i*th simulated variable, *X_obs_^i^*^th^ is the *i*th measured variable, *n* the sample size, and $\bar{X_{obs}}$ the average observed variable.

### 3.1. Results of the Sensitivity Analysis

**Figure 2:**
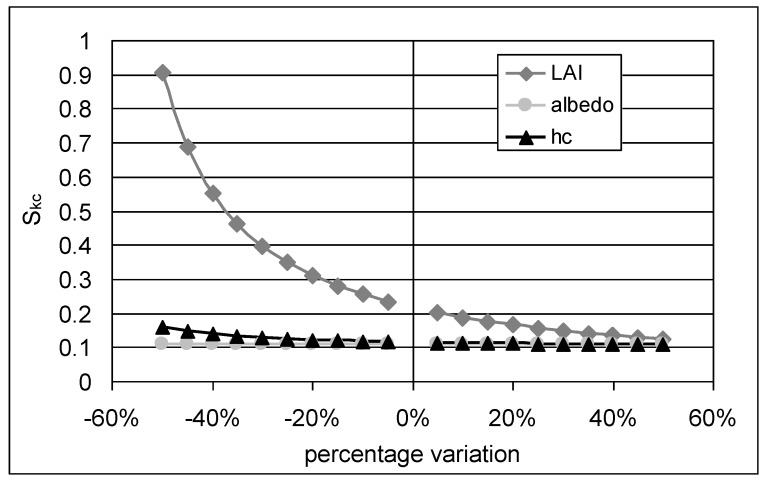
Sensitivity coefficient of kc at LAI, albedo and vegetation height variations.

In Figure 2 the sensitivity coefficients for kc are reported for LAI, albedo and vegetation height considering a variation from the mean value of ±50%, using the meteorological variables to compute energy fluxes measured by an eddy covariance station located in a maize field in Northern Italy [50].

Albedo and vegetation height have a low influence on crop coefficient estimates; in fact *S_Kc_* remains almost constant and equal to 0.1 for variations of albedo and vegetation height from −50% to +50%. Instead the crop coefficient is highly influenced by the variability of leaf area index and in particular decreases in exponential way from −50% variation of LAI with *S_Kc_* equal to 0.44 till values of 0.13 with a variation of +50% of LAI, so that the crop coefficient will be more sensitive during the growing period of the vegetation. These results are also confirmed by [51] who found that the most sensitive parameter in *ET*_0_ estimation is *r_c_*.

### 3.2. Pasture: Torgnon

**Figure 3:**
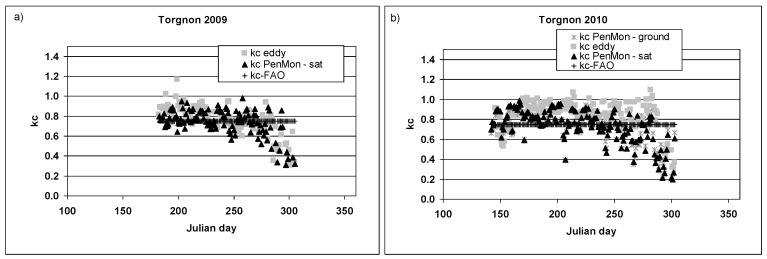
Crop coefficient values for Torgnon eddy covariance station over pasture for: (**a**) 2009; and (**b**) 2010.

In Figure 3 the computed crop coefficient values are reported for 2009 and 2010. The selected data belong to snow-free periods from day 183 to 305 for 2009 and from 142 to 302 for 2010. Results for kc are summarized in Table 1, while in Table 2 the lengths of the growth stages are reported.

During the year 2009, two crop coefficient values from eddy covariance measurements can be identified: the first one is relative to a completely developed grass with a mean values of 0.8, while the second one is relative to the senescence phase when grass starts to become yellow with a mean value of 0.47. A similar distribution is found also during 2010, with the first *kc_eddy_* equal to 0.88 and the second to 0.43.

When the crop coefficient is computed with Penman-Monteith’s equation, a *kc_PenMon,ground_* of 0.76 is obtained during 2010 for the first period and 0.39 for the senescence period. These values are quite similar to those obtained from eddy covariance data, showing that for pasture correct kc values can be defined using meteorological data from basic network stations which are more available than from eddy covariance towers.

The analyses are then performed using satellite information of LAI and *kc_PenMon,sat_* are equal to 0.76 for the first period and 0.49 for the second period during 2009, while 0.76 and 0.36 for 2010. *kc_PenMon,sat_* are similar to *kc_PenMon,ground_* and *kc_eddy_*, showing that this technique can be applied in a distribute way in river basins coupling basic network stations for meteorological data and satellite data for vegetation information.

Finally, RMSE values have been computed between *kc_PenMon,sat_* and *kc_eddy_* and RMSE values equal to 0.15 and 0.19 are found for 2009 and 2010 respectively. When *kc_PenMon,ground_* and *kc_eddy_* are compared for 2010, a RMSE of 0.18 is obtained.

### 3.3. Deciduous Forest

#### 3.3.1. Chestnut Ridge

**Figure 4:**
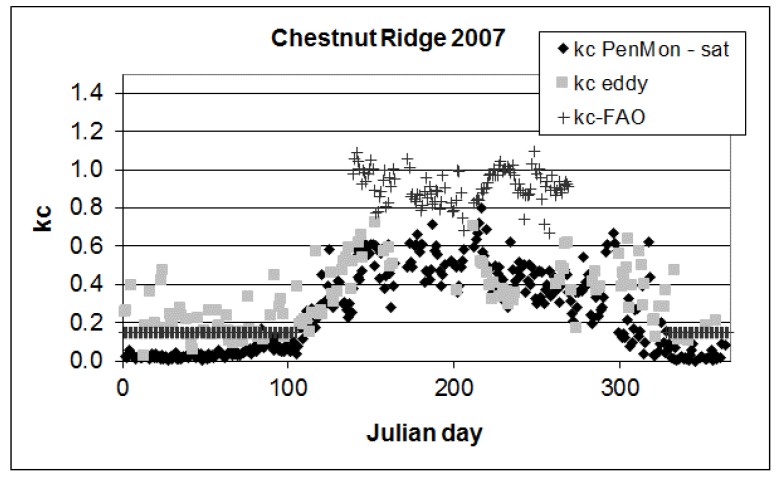
Crop coefficient values for Chestnut Ridge eddy covariance station for 2007.

In Figure 4 crop coefficient values are reported for the deciduous forest of Chestnut Ridge. The data belong to the whole year, so that the complete seasonal cycle can be analyzed differencing the behaviors during periods with or without leaves (Table 1). In Table 2 the lengths of the growth stages are reported.

During the winter period, *kc_eddy_* has low values equal to 0.19. Similar results are obtained during the autumn season when the leaves are not present as well. kc from eddy covariance tower is then equal to 0.2. When these periods are analyzed using satellite information, much lower values are obtained with *kc_PenMon,sat_* for the initial period equal to 0.04 and *kc_-end_* to 0.05 for both years. This behaviour is linked to values near zero or zero of LAI from satellite during these winter–autumn periods.

The growth and decrease branches of the two kc curves are quite in accordance, according to Figure 4, as well as for the summer period when *kc_-mid_* from eddy covariance station is equal to 0.47 and from satellite data to 0.48. Overall, analyzing the estimates accuracy for the whole year, RMSE obtained from the comparison between kc computed from eddy covariance data and from satellite data is found to be equal to 0.18.

#### 3.3.2. Duke Forest

**Figure 5:**
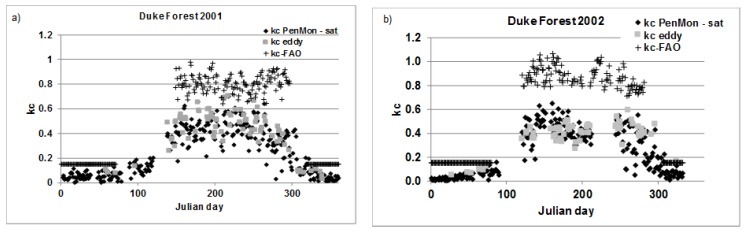
Crop coefficient values for Duke forest eddy covariance station for: (**a**) 2001; and (**b**) 2002.

Crop coefficient results for the years 2001 and 2002 are reported in Figure 5. *kc_eddy_* during 2001 is low during winter with a mean value of 0.11 while *kc_PenMon,sat_* is 0.07. Similar values are obtained for 2002 with *kc_PenMon,sat_* being 0.04 and *kc_eddy_* 0.09. These results are obtained also during autumn (Table 1). During the summer season, when trees are completely lush, as expected, crop coefficient values are higher and equal to 0.51 from eddy covariance data and to 0.44 from satellite data for 2001; while for 2002 kc values of 0.43 and 0.44 are reached for *kc_eddy_* and *kc_PenMon,sat_* respectively. Results for the two analyzed year are quite in accordance with each other. The lengths of the growth stages are similar between the two analyzed years (Table 2).

When the yearly RMSE is evaluated between kc from eddy covariance data and from Penman-Monteith’s equation with satellite data, values of 0.05 for 2001 and of 0.04 for 2002 are obtained.

#### 3.3.3. Intercomparison

A comparison between Chestnut Ridge and Duke Forests results is performed in order to define a general crop coefficient curve which is representative for deciduous forests. According to Table 1, results of crop coefficient from eddy covariance data are quite similar between the two stations for the initial stage value for all the analysed year ranging between 0.09 and 0.19, as well as for the final stage with values between 0.12 and 0.2. *kc_-mid_* is also quite defined with values between 0.43 and 0.48.

### 3.4. Evergreen Forest: Black Hills

**Figure 6:**
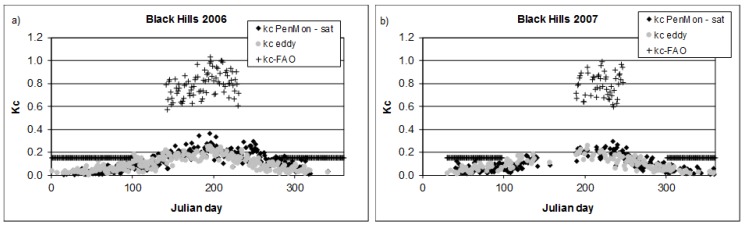
Crop coefficient values for Black Hills forest eddy covariance station for: (**a**) 2006; and (**b**) 2007.

In Figure 6 crop coefficient values are reported for 2006 and 2007 for the Black Hills station. Even though the station is located in an evergreen forest, evapotranspiration substantially decreases during the winter period and so the crop coefficient does.

In Table 1 *kc_eddy_* has low values equal to 0.05 during the winter period for both years. Similar valus are obtained for *kc_PenMon,sat_*. The final stage has similar low values equal to 0.03 and 0.07 during 2006 from eddy covariance data and from satellite data respectively, while for 2007 *kc_eddy_* is equal to 0.03 while *kc_PenMon,sat_* to 0.04.

The growth and decrease branches of the two kc curves are quite in accordance, according to Figure 4 and Table 2, as well as for the summer period when *kc_-mid_* from eddy covariance station is equal to 0.17 and from satellite data to 0.2 for 2006 and to 0.18 and 0.2 respectively for 2007. Overall, analyzing the estimates accuracy for the whole year, RMSE between kc from eddy covariance data and from satellite data is equal to 0.07 in 2006 and to 0.05 in 2007.

## 4. Conclusions

In this paper, the potentiality of using evapotranspiration measurements from micro-meteorological stations for definition of crop coefficient functions of natural vegetated area has been evaluated. Sensitivity analysis shows that albedo and vegetation height have a low influence on crop coefficient estimates, while these are highly influenced by the variability of leaf area index.

For pasture, crop coefficient obtained from eddy covariance data are similar to the values obtained with ground measured data and those obtained using leaf area index and albedo retrieved from remotely sensed images. This leads to two important results: (1) measurements from widespread available stations provide robust estimate of crop coefficient, and (2) satellite data are powerful to compute crop coefficient in a distribute way in river basins wherever basic ground measured meteorological data are available.

The potentials of remotely sensed data have been confirmed using data available from the FLUXNET network both on deciduous and evergreen forests. The crop coefficient values for the considered coverage of pastures, deciduous and evergreen forests are found to be lower than the FAO values during the mid-season stage, showing the necessity to check FAO data before applying them to areas with climatic and vegetation characteristics different form the ones considered by FAO.

## Figures and Tables

**Table 1:** Crop coefficient values for the four sites: *kc_eddy_*, *kc_PenMon,sat_*, *kc_PenMon,ground_*, *kc_FAO_*.

			*kc_-ini_*	*kc_-mid_*	*kc_-end_*
**Pasture**					
**Torgnon**		***kc_FAO_***	0.75	0.75	0.75
	**2009**	***kc_eddy_***	-	0.8	0.47
		*kc_PenMon,sat_*	-	0.76	0.49
	**2010**	***kc_eddy_***	-	0.88	0.43
		*kc_PenMon,sat_*	-	0.76	0.36
		*kc_PenMon,ground_*	-	0.76	0.39
**Deciduous Forest**					
**Chestnut Ridge**	**2007**	***kc_FAO_***	0.15	0.91	0.15
		***kc_eddy_***	0.19	0.47	0.2
		*kc_PenMon,sat_*	0.04	0.48	0.05
**Duke Forest**	**2001**	***kc_FAO_***	0.15	0.8	0.15
		***kc_eddy_***	0.11	0.51	0.12
		*kc_PenMon,sat_*	0.07	0.44	0.06
	**2002**	***kc_FAO_***	0.15	0.9	0.15
		***kc_eddy_***	0.09	0.43	-
		*kc_PenMon,sat_*	0.04	0.44	0.07
**Evergreen Forest**					
**Black Hills**	**2006**	***kc_FAO_***	0.15	0.79	0.15
		***kc_eddy_***	0.05	0.17	0.04
		*kc_PenMon,sat_*	0.04	0.20	0.07
	**2007**	***kc_FAO_***	0.15	0.78	0.15
		***kc_eddy_***	0.05	0.18	0.03
		*kc_PenMon,sat_*	0.05	0.20	0.04

**Table 2:** Lengths of crop development stages (Julian day of beginning and end) respect to *kc_-ini_*, *kc_-mid_* and *kc_-end_*.

		Days *kc_-ini_*	Days *kc_-mid_*	Days *kc_-end_*
**Pasture**				
**Torgnon**	2009		183–305	
	2010		143–303	
**Deciduous Forest**				
**Chestnut Ridge**	2007	1–105	139–269	328–365
**Duke Forest**	2001	1–71	149–297	320–365
	2002	1–80	121–281	306–365
**Evergreen Forest**				
**Black Hills**	2006	1–98	142–231	281–365
	2007	1–98	189–247	301–365
